# Predicting the Swallow-Related Quality of Life of the Elderly Living in a Local Community Using Support Vector Machine

**DOI:** 10.3390/ijerph16214269

**Published:** 2019-11-03

**Authors:** Haewon Byeon

**Affiliations:** Department of Speech Language Pathology, School of Public Health, Honam University, 417, Eodeung-daero, Gwangsan-gu, Gwangju 62399, Korea; bhwpuma@naver.com; Tel.: +82-10-7404-6969

**Keywords:** swallowing quality-of-life, dysphagia, elderly living in a local community, support vector machine, risk factor

## Abstract

*Background and Objectives:* This study developed a support vector machine (SVM) algorithm-based prediction model with considering influence factors associated with the swallowing quality-of-life as the predictor variables and provided baseline information for enhancing the swallowing quality of elderly people’s lives in the future. *Methods and Material:* This study sampled 142 elderly people equal to or older than 65 years old who were using a senior welfare center. The swallowing problem associated quality of life was defined by the swallowing quality-of-life (SWAL-QOL). In order to verify the predictive power of the model, this study compared the predictive power of the Gaussian function with that of a linear algorithm, polynomial algorithm, and a sigmoid algorithm. *Results:* A total of 33.9% of the subjects decreased in swallowing quality-of-life. The swallowing quality-of-life prediction model for the elderly, based on the SVM, showed both preventive factors and risk factors. Risk factors were denture use, experience of using aspiration in the past one month, being economically inactive, having a mean monthly household income <2 million KRW, being an elementary school graduate or below, female, 75 years old or older, living alone, requiring time for finishing one meal on average ≤15 min or ≥40 min, having depression, stress, and cognitive impairment. *Conclusions:* It is necessary to monitor the high-risk group constantly in order to maintain the swallowing quality-of-life in the elderly based on the prevention and risk factors associated with the swallowing quality-of-life derived from this prediction model.

## 1. Introduction

A swallowing problem is one of the frailty symptoms caused by aging and it can occur in healthy elderly people due to a missing tooth, esophageal weakness, and decreased cognitive function [1,2]. A nationwide survey of South Korea [3] reported that, as of 2014, 54.6% of the elderly population had difficulties in conducting daily activities due to a swallowing problem caused by the decrease in chewing ability. If a swallowing problem persists, it is highly likely to cause dysphagia. Therefore, it may decrease the ability to eat and drink efficiently and result in secondary problems such as lack of nutrient intake, dehydration, and deteriorated body functions. In severe cases, it can lead to death due to aspiration pneumonia [4]. Additionally, since dysphagia is known to decrease the quality of elderly people’s lives [5], identifying factors affecting the swallowing quality-of-life in rehabilitation science is an important topic.

Previous studies have identified old age, gender, cognitive level, stress, number of missing teeth, resilience, and social confidence as factors affecting the swallowing quality-of-life [2,6]. Roy et al. [7] reported that “taking a longer time to eat”, “a sensation of food stuck in the throat”, and “coughing, throat clearing, or choking before, during, or after eating” were major risk factors of swallowing disorders. Byeon [8] also showed that older adults using dentures had a 1.6-fold higher risk of a swallowing disorder and those accompanied by physical dysfunction had a 4.3-fold higher risk of a swallowing disorder than healthy elderly people.

However, these studies only identified individual risk factors because (1) they only focused on dysphagia-induced physical problems in patients with the damaged central nervous system such as stroke [9], and (2) they mainly used either a regression model or general linear model to evaluate factors associated with the swallowing quality-of-life [2,6,7,8]. Therefore, it is very meaningful to identify multiple risk factors influencing the swallowing quality-of-life, such as sociodemographic factors, and establishing measures to enhance the swallowing quality-of-life based on the findings.

Recent medical and public health studies have utilized data mining techniques, such as support vector machine (SVM), to explore complex risk factors of diseases [10]. SVM is less likely to cause an over-fitting problem than decision trees and it is also possible to classify non-linear data when using a kernel method, which is an advantage [11]. This study developed an SVM algorithm-based prediction model with considering various influence factors associated with the swallowing quality-of-life (e.g., sociodemographic variables, cognitive function, stress, and depression) as the predictor variables and provided baseline information for enhancing the swallowing quality of elderly people’s lives in the future.

## 2. Subjects and Methods

This was a cross-sectional study to identify factors affecting the swallowing quality-of-life and it targeted elderly people aged 65 years or over. This study was approved by the Institutional Review Board of Honam University (No.1041223-201812-HR-26) and was conducted in accordance with the ethical standards of the Declaration of Helsinki. This study sampled elderly people who were using a senior welfare center located in Seoul, Incheon, or Suwon using a convenience sampling method. Sample selection criteria were (1) those who did not have a history of a neurological disease (e.g., stroke or Parkinson’s disease) that could affect swallowing; (2) those who scored at least 20 points in the Korean version of the Mini-Mental State Examination (MMSE-K), indicating no dementia; and (3) those who agreed to participate in the study. This study estimated the required number of subjects using G*Power ver. 3.1.9.3 at effect size = 0.15, significance level = 0.05, power = 0.95, and number of predictors = 12. It was estimated that the study would need at least 132 subjects. This study recruited 150 subjects on the assumption of a 10% dropout rate, and analyzed data from 142 subjects after excluding subjects who requested to drop out during the questionnaire.

The swallowing problem associated with quality-of-life was defined by the swallowing quality-of-life (SWAL-QOL) [12]. The SWAL-QOL is composed of 44 items under eleven sub-divisions: Two items of ‘pressure’, two items of ‘eating time’, three items of ‘appetite’, 14 items of ‘frequency of symptoms’, two items of ‘food choice’, two items of ‘communication’, four items of ‘fear’, five items of ‘mental health’, five items of ‘social functions’, three items of ‘fatigue’, and two items of ‘sleep’. Each item was measured by the five-point Likert scale (strongly agree = 1, and strongly disagree = 5). The total score ranges from 44 to 220 points and a higher score indicates better swallowing quality-of-life. This study used 111 points as a cut-off score referring to previous studies [13,14] on South Korean elderly people. Cronbach’s α, indicating the internal consistency, was 0.93.

The cognitive level was defined by MMSE-K [15]. MMSK-K is composed of 30 points, and ≤23 was classified as cognitive impairment. Cronbach’s α was 0.88. Depression was measured by the geriatric depression scale short form Korea version (GDSSF-K) [16]. The total score of it is 15 points, and a higher score means a severe depression level. Depression was defined as a score ≥ 5. Cronbach’s α was 0.89. Life stress was measured using Seo’s elderly stress scale (SESS) [17]. SESS consists of 23 items: ‘economic stress (6 items)’, ‘health stress (8 items)’, ‘family stress (9 items)’. Each item is measured by a 5-point Likert scale (strongly disagree = 1, and strongly agree = 5). A higher score indicates more severe life stress, and this study defined ≥70 as the presence of stress. Cronbach’s α was 0.85.

The sociodemographic factors of this study included age (65–74 years old or ≥75 years old), gender, education level (elementary school graduate and below, middle school graduate, or high school graduate or above), living with a family (living with a spouse and a child, living only with a spouse, living only with a child, and living alone), economy activity (yes or no), mean monthly household income (<2 million KRW, 2–4 million KRW, or >4 million KRW), experience of aspiration in the past 1 month (yes or no), mean required time to finish a meal (≤15 min, 16–39 min, or ≥40 min), and denture use (yes or no).

**Figure 1:**
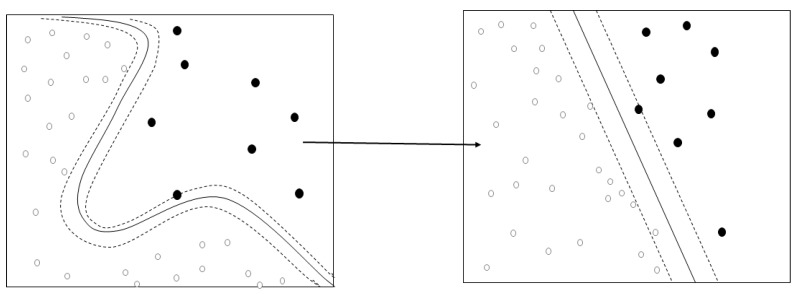
Concepts of kernel algorithm.

The SVM was used to develop a model to predict the swallowing quality-of-life of the elderly living in a local community (Figure 1). The SVM is a machine-learning algorithm searching for the optimal decision boundary [18]. In other words, it is a linear discriminant function that transforms learning data to a higher dimension through non-linear mapping and optimally separates hyperplane. For example, if A = (a, d) and B = (b, c) are non-linearly separable in the two-dimension, they can have linearly separable characteristics when they are mapped in three-dimensions. Therefore, when appropriate nonlinear mapping is used in a sufficiently large dimension, data containing two classes can always be separated in hyperplanes [19].

## 3. Results

### 3.1. General Characteristics of Subjects

The general characteristics of 142 subjects were analyzed and summarized (Table 1). The majority of the subjects was equal to or older than 75 years old (79.2%). In total, 78.3% of the subjects were female and 21.7% were male. A total of 59.0% of the subjects were elementary school graduate level or below, 42.2% lived alone, 10.1% of the subjects were economically inactive, 72.8% of households had less than 2 million KRW monthly, 62.2% of the subjects swallowed the wrong way in the past one month, 63.7% used dentures, and 66.5% of the subjects spent 16–39 min for a meal on average. A total of 17.7% of subjects had depression, 20.5% of the subjects recognized stress, 28.0% of the subjects had a cognitive impairment, and 33.9% of the subjects decreased in swallowing quality-of-life.

### 3.2. The General Characteristics of Subjects According to the Level of the Swallowing Quality-of-Life

The general characteristics of subjects according to the level of the swallowing quality-of-life and the factors potentially related to the swallowing quality-of-life are shown in Table 2. The results of the Chi-square test showed that the swallowing quality-of-life was significantly affected by age, gender, education level, living with a family or not, mean required time for completing a meal, aspiration experience in the past one month, denture use, and cognitive level. The proportion of the elderly with a low swallowing quality-of-life was significantly higher when the subjects were equal to or older than 75 years old (38.4%), were female (44.6%), were elementary school graduate or below (35.7%), were living alone (31.7%), experienced aspiration in the past one month (39.8), took 40 min or more to finish one meal on average (40.0%), used dentures (40.0%), and had cognitive impairment (45.0%).

### 3.3. The Function Weights of Gaussian Kernel Algorithm-Based SVM

The function weights of the Gaussian kernel algorithm-based SVM are shown in Table 3. Although function weights of the SVM are not for simply comparing the sizes of variables or the importance of variables, it is possible to identify whether the relationship between a predictor and an outcome variable is risk or prevention. The swallowing quality-of-life prediction model for the elderly based on the SVM showed both preventive factors and risk factors. Risk factors were denture use, experience of using aspiration in the past one month, being economically inactive, having a mean monthly household income <2 million KRW, being an elementary school graduate or below, female, 75 years old or older, living alone, requiring time for finishing one meal on average ≤15 min or ≥40 min, suffering depression, stress, and cognitive impairment. On the other hand, preventive factors for those aged between 65 and 74 years old included being a middle school graduate or above, having a mean monthly household income ≥2 million KRW, no experience of aspiration in the past one month, requiring time for finishing one meal on average between 16 and 30 min, not using dentures, being economically active, not suffering depression or stress, and having a normal cognitive level. The final prediction rate of the SVM using 435 support vectors was 91.08.

### 3.4. The Prediction Accuracy of the SVM-Based Swallowing Quality-of-Life

The prediction accuracy of the SVM-based swallowing quality-of-life classification algorithm is shown in Table 4. In the SVM, the fitness of a model varies by the type of a kernel. Therefore, this study compared the prediction accuracy of the linear, polynomial, and sigmoid algorithms and the Gaussian Kernel in order to compare the performance of a model depending on various kernel types. Moreover, the SVM types of the four algorithms were divided into C-SVM and Nu-SVM SVM types to compare their prediction accuracies. The fitness results showed that the accuracy of C-SVM and that of Nu-SVM did not differ much in this swallowing quality-of-life prediction model. Moreover, it was found that the accuracy of the Gaussian kernel was highest, while that of the sigmoid kernel was lowest.

## 4. Discussion

This study developed an SVM-based swallowing quality-of-life prediction model for elderly people living in a local community. The results of this study showed that 33.9% of the elderly had low swallowing quality-of-life. Although it is not possible to compare directly, the results are similar to the results of the previous studies [7,8,20,21] conducted in the US and Japan, which reported that the proportion of dysphagia risk group ranged from 33% to 52.6% when they stayed at home. In many cases, the elderly living at home tended to consider the symptoms related to swallowing as inevitable symptoms in the process of aging, although they experienced and were aware of these symptoms, and they mostly did not even perceive that they needed to receive appropriate evaluation or treatment [8]. Particularly, the results of this study revealed that one of three elderly people living at home had low swallowing quality-of-life even though they could live independently without physical limitations. The results implied that it would be necessary to monitor the swallowing function of elderly people living in a local community continuously.

In this study, denture use, aspiration experience in the past one month, being economically inactive, having a mean monthly household income <2 million KRW, being an elementary school graduate or below, female, 75 years old or older, living alone, requiring time for finishing one meal on average ≤15 min or ≥40 min, having depression, stress, and cognitive impairment were predictors associated with the swallowing quality-of-life. A number of studies explored the risk factors of dysphagia and reported that sociodemographic factors (e.g., gender, age, and education level), denture use, and mean required time to finish a meal on average were factors influencing the swallowing quality-of-life [2,6,7], and it supported the results of this study.

In particular, the cognitive level is known to be closely associated with dysphagia. Elderly people with a decreased cognitive function cannot maintain a normal harmony among oral, pharyngeal, and laryngeal movements due to the changes in tongue movements, the rise or contraction of the hyoid bone and upper esophageal sphincter, or decreased sensory functions [22]. Consequently, the swallowing problem occurs due to decreased coordination [22,23]. Chouinard (2000) also reported that the quality of dementia patients’ lives was affected by a swallowing problem. For example, it was reported that dementia elderly had a swallowing problem due to the characteristic of delaying swallowing in the pharynx in the early stage, and it became more difficult to swallow food in the mouth as dementia became more severe [22]. Since a swallowing program can cause serious complications such as aspiration pneumonia or malnutrition when it persists for a long time, it is required to monitor a high-risk group continuously and develop a systematic guideline associated with dysphagia in order to prevent the risk of complications and maintain good swallowing quality-of-life.

Another finding of this study was that denture use was associated with low swallowing quality-of-life for the elderly. Byeon [8] identified the risk factors of the dysphagia for the elderly living in a local community using a cross-sectional approach and reported that elderly people using dentures had a 1.6-times higher risk of experiencing dysphagia than those not using dentures. It is believed that elderly people using dentures have a low swallowing quality-of-life because the dentures decrease the secretion of saliva and interfere with the formation of the bolus to adversely affect food intake [24]. Although it is highly possible that elderly people using dentures experience a decrease in swallowing quality-of-life, education programs for dysphagia have been conducted mainly for hospitalized patients in a medical institute and programs for the elderly living at home are very rare [25]. Therefore, it is necessary to develop a program customized for the elderly using dentures that can preserve the swallowing function.

This study compared the prediction accuracy of eight SVM classification algorithms (four algorithms—Gaussian kernel, linear kernel, polynomial kernel, and sigmoid, and two SVMs—C-SVM and Nu-SVM) in order to compare the performance of the SVM according to algorithms. It was found that the prediction accuracy of the C-SVM Gaussian kernel was the highest. The performance of the non-linear SVM largely depends on the kernel function and its parameters [19]. The Gaussian kernel is an algorithm that maps something in a specific space with infinite dimensions and previous studies [26,27] reported that it is an algorithm with high prediction accuracy. The results of this study suggested that using Gaussian kernel-based C-SVM is effective in predicting binary disease/impairment data.

The importance of this study is that it identified factors influencing the swallowing quality-of-life of the elderly living in a local community; these included cognitive function, depression, stress, demographic variables, and physical problems. The limitations of the study are as follows. First, this study did not examine the swallowing function levels of the subjects, which are related to the swallowing quality-of-life. It is necessary to develop a swallowing quality-of-life prediction model by conducting a standardized dysphagia risk assessment, such as the modified dysphagia risk assessment scale for an elderly person (DRACE) [24]. Second, it is not possible to generalize the results of this study because the study subjects were recruited by a convenience sampling method. Future studies are needed to conduct a survey by using a systematic sampling, such as a random sampling. Third, the results of this study cannot be interpreted as a causal relationship because this study analyzed cross-sectional data. A longitudinal study is necessary to demonstrate the causality of the risk factors identified in this study in the future.

## 5. Conclusions

The results of this study can be used as a basis to establish strategies for preventing and managing the swallowing problem of the elderly living in the community. It is necessary to monitor the high-risk group constantly in order to maintain the swallowing quality-of-life in the old age, based on the prevention and risk factors associated with the swallowing quality-of-life derived from this prediction model. Furthermore, it is needed to develop a customized education program for the elderly living in a local community to prevent dysphagia.

## Figures and Tables

**Table 1:** General characteristics of subjects, *n* (%).

Variables	Subcategory	Total (*n* = 142)
Age	65–74	30 (20.8)
	≥75	112 (79.2)
Gender	Male	31 (21.7)
	Female	111 (78.3)
Education level	Elementary school graduate and below	84 (59.0)
	Middle school graduate	29 (20.5)
	High school graduate or above	29 (20.5)
Living with a family	Living with a spouse and a child	31 (22.1)
	Living only with a spouse	28 (19.5)
	Living only with a child	23 (16.2)
	Living alone	60 (42.2)
Economy activity	Yes	14 (10.1)
	No	128 (89.9)
Mean monthly household income	<2 million KRW	103 (72.8)
	2–4 million KRW	27 (18.7)
	>4 million KRW	12 (8.5)
Experience of aspiration in the past 1 month	Yes	88 (62.2)
	No	54 (37.8)
Mean required time to finish a meal	≤15 min	43 (30.3)
	16–39 min	94 (66.5)
	≥40 min	5 (3.2)
Denture use	Yes	90 (63.7)
	No	52 (36.3)
Cognitive level	Normal	102 (72.0)
	Cognitive impairment	40 (28.0)
Depression	Yes	25 (17.7)
	No	117 (82.3)
Life stress	Yes	29 (20.5)
	No	113 (79.5)
Swallowing Quality-of-Life	High	94 (66.1)
	Low	48 (33.9)

**Table 2:** The factors potentially related to the swallowing quality-of-life, %.

**Variables**	**Subcategory**	**Swallowing-Quality of Life**		***p***
		**Low (*n* = 48)**	**High (*n* = 94)**	
Age	65–74	9 (30.0)	21 (70.0)	<0.001
	≥75	43 (38.4)	69 (61.6)	
Gender	Male	10 (32.3)	21 (67.7)	<0.001
	Female	45 (44.6)	56 (55.4)	
Education level	Elementary school graduate and below	30 (35.7)	54 (64.3)	<0.001
	Middle school graduate	9 (31.0)	20 (69.0)	
	High school graduate or above	6 (20.7)	23 (79.3)	
Living with a family	Living with a spouse and a child	5 (16.1)	26 (83.9)	<0.001
	Living only with a spouse	6 (17.6)	28 (82.4)	
	Living only with a child	5 (17.9)	23 (82.1)	
	Living alone	19 (31.7)	41 (68.3)	
Economy activity	Yes	4 (28.6)	10 (71.4)	0.415
	No	39 (30.5)	89 (69.5)	
Mean monthly household income	<2 million KRW	20 (19.4)	83 (80.6)	0.153
	2–4 million KRW	5 (18.5)	22 (81.5)	
	>4 million KRW	2 (16.7)	10 (83.3)	
Experience of aspiration in the past 1 month	Yes	35 (39.8)	53 (60.2)	<0.001
	No	12 (22.2)	42 (77.8)	
Mean required time to finish a meal	≤15 min	13 (30.2)	30 (69.8)	<0.001
	16–39 min	10 (10.6)	84 (89.4)	
	≥40 min	2 (40.0)	3 (60.0)	
Denture use	Yes	36 (40.0)	54 (60.0)	<0.001
	No	11 (21.2)	41 (78.8)	
Cognitive level	Normal	13 (12.7)	89 (87.3)	<0.001
	Cognitive impairment	18 (45.0)	22 (55.0)	
Depression	Yes	5 (20.0)	20 (80.0)	0.583
	No	24 (20.5)	93 (79.5)	
Life stress	Yes	8 (8.5)	86 (91.5)	0.830
	No	4 (8.3)	44 (91.7)	

**Table 3:** The function weights of Gaussian kernel algorithm.

65–74 years old	−0.008
≥75 years old	0.017
Male	−0.011
Female	0.015
Elementary school graduate and below	0.019
Middle school graduate	−0.007
High school graduate or above	−0.030
Living with a spouse and a child	−0.018
Living only with a spouse	−0.011
Living only with a child	−0.007
Living alone	0.008
Economy activity	0.011
Economy inactivity	−0.031
Mean monthly household income: <2 million KRW	0.029
Mean monthly household income: 2–4 million KRW	−0.015
Mean monthly household income: >4 million KRW	−0.021
Experience of aspiration in the past 1 month: Yes	0.054
Experience of aspiration in the past 1 month: No	−0.009
Mean required time to finish a meal: ≤15 min	0.034
Mean required time to finish a meal: 16–39 min	−0.011
Mean required time to finish a meal: ≥40 min	0.023
Denture use: Yes	0.045
Denture use: No	−0.030
Cognitive level: Normal	−0.009
Cognitive impairment	−0.028
Depression: Yes	0.005
Depression: No	−0.003
Life stress: Yes	0.011
Life stress: No	−0.019
Number of Support Vector: 435	

**Table 4:** The prediction accuracy of the SVM-based swallowing quality-of-life, %.

Type of SVM	Type of Kernel			
	Linear	Polynomial	Gaussian	Sigmoid
C-SVM	90.95	90.31	91.08	89.75
Nu-SVM	90.43	90.28	91.03	89.66
